# The Vital Role of Clinical Examination in Unmasking Bell's Palsy: Beyond Pattern Recognition

**DOI:** 10.7759/cureus.55311

**Published:** 2024-03-01

**Authors:** Ahmad B Abdelrehim, Salman Kananeh, Daniel Landau

**Affiliations:** 1 Internal Medicine, Capital Health Regional Medical Center, Trenton, USA; 2 Internal Medicine Residency Program, Capital Health Regional Medical Center, Trenton, USA; 3 Neurology, Capital Health Regional Medical Center, Trenton, USA

**Keywords:** neuro deficits, neuro-imaging, facial palsy, bell`s palsy, s: multiple sclerosis

## Abstract

While multiple sclerosis (MS) commonly manifests with optic nerve involvement, it can also masquerade as diverse cranial nerve (CN) palsies. We present the case of a young male initially diagnosed with Bell's palsy based on unilateral facial nerve paralysis. Despite the presence of typical clinical features, the patient's evaluation took an unexpected turn. Subsequent brain MRI revealed demyelinating lesions, ultimately confirming the diagnosis of MS. This case underscores the importance of maintaining vigilance in diagnosing atypical presentations of MS, illustrating how meticulous evaluation and neuroimaging play pivotal roles in uncovering underlying pathologies when conventional diagnoses such as Bell's palsy raise uncertainties.

## Introduction

Bell’s palsy is the primary cause of acute unilateral facial paralysis, predominantly affecting the lower motor neurons of the facial nerve. This condition typically manifests as sudden facial weakness, gradually resolving on its own in approximately 80-90% of cases [1,2]. The etiology of Bell's palsy is multifactorial, with suspected triggers including various viral infections such as herpes simplex virus (HSV), herpes zoster, Lyme disease, syphilis, Epstein-Barr virus, cytomegalovirus, HIV, and mycoplasma. Additionally, conditions like diabetes mellitus and hypertension, leading to inflammation and microvascular dysfunction, have been implicated as potential causative factors [1,2]. Pathologically, the lesion in Bell's palsy mainly localizes around the geniculate ganglion within the facial canal. Nonetheless, central lesions at the level of the ipsilateral facial nucleus or facial nerve within the pons can also contribute to its manifestation [3].

Conversely, multiple sclerosis (MS) is a chronic autoimmune inflammatory disorder characterized by demyelination and subsequent axonal degeneration within the central nervous system (CNS). While the optic nerve is commonly involved in MS-related cranial nerve (CN) issues, MS can also manifest with or initially present as other CN palsies, adding complexity to its diagnostic landscape [4]. We present a compelling case of a young male patient initially diagnosed with Bell’s palsy based on classical clinical features of unilateral facial nerve paralysis. However, meticulous evaluation, including neuroimaging via brain MRI, threw up an unexpected revelation, ultimately leading to the diagnosis of MS [5]. This case report emphasizes the crucial need for a comprehensive assessment when encountering atypical presentations of CN palsies, highlighting the pivotal role of neuroimaging in revealing underlying pathologies that might deviate from conventional diagnoses such as Bell's palsy.

## Case presentation

Medical history

A 22-year-old previously healthy male sought emergency medical attention for left-sided facial drooping that had developed over the past seven days, which was accompanied by episodes of dizziness while walking and double vision when looking left, without any reported falls. Additionally, the patient had experienced two distinct episodes of vomiting in the two days preceding his ED visit, with no associated diarrhea. He had also noted an altered taste perception localized to the left side while eating.

Two weeks before the onset of facial symptoms, the patient had experienced frontal throbbing headaches along with a sore throat, which had tested negative for streptococcal infection. Despite subjective reports of fever and chills, he denied any auditory symptoms like hearing loss or tinnitus, as well as any complaints of weakness or numbness in other body regions, cough, rhinorrhea, chest pain, or changes in urination.

Examination

During examination, the patient appeared comfortable without any acute distress, yet exhibited noticeable right-sided deviation of the mouth, without any apparent facial rash. There were no indications of jugular venous distention (JVD), and auscultation revealed clear chest sounds with normal S1-S2 and a relaxed abdomen. In the ear assessment, bilateral findings were within normal limits. The external auditory canals were patent, and devoid of obstructions or discharge. Inspection of the tympanic membranes revealed intact and translucent membranes, displaying a pearly gray coloration with a visible cone of light in the anterior inferior quadrant.

Further CN examination unveiled flattening of the forehead and nasolabial fold on the left side, with attempted forehead wrinkling leading to asymmetry. Smiling resulted in deviation of the mouth to the right side, while attempted eye closure showed upward rolling of the left eye. Pupillary examination indicated medium-sized, reactive pupils, while eye movement assessment revealed impaired adduction of both eyes, accompanied by lateral-beating nystagmus, suggestive of internuclear ophthalmoplegia, along with bilateral upward-beating nystagmus. A weakened left corneal reflex and reported loss of taste sensation over the left side of the tongue were noted. Motor examination demonstrated 5/5 strength in all extremities with intact reflexes, and the sensory system was grossly intact.

Laboratory findings

Table 1 displays the results of basic laboratory investigations. Serum ACE level was 39 mcg/L (normal: <40), and plasma ANA yielded a negative result. Serological tests showed negative results for Lyme disease, West Nile virus IgM, and VDRL, while varicella-zoster virus IgM was positive with a low titer. Additionally, HSV 1 IgG was detected at a level of 25-30, whereas HSV 2 IgG tested negative.

**Table 1:** Laboratory investigations *Traumatic CSF tap which became clearer with consecutive CSF tube WBCs: white blood cells; BUN: blood urea nitrogen; AST: aspartate aminotransferase, ALT: alanine aminotransferase; ALP: alkaline phosphatase; CSF: cerebrospinal fluid; MBP: myelin basic protein

Tests	Result	Normal range
Blood/plasma		
WBCs, x 10^3^/mm^3^	10.6	4-11
Hemoglobin, g/dl	14.6	Male: 13.5-17.5
Platelets, x 10^3^/mm^3^	327	150-450
Sodium, mEq/l	139	135-150
Potassium, mEq/l	3.7	3.5-5
Chloride, mEq/l	99	96-106
Bicarbonate, mmol/l	29	23-29
BUN, mg/dl	15	6-20
Creatinine, mg/dl	0.2	0.6-1.3
AST, U/L	23	8-33
ALT, U/L	21	4-36
ALP, U/L	62	20-130
Calcium, mg/dl	10.5	8.5-10.2
Glucose, mg/dl	101	70-100
Urine drug screen	Negative	
CSF		
WBC, cell/mm^3^	4-9	<5
RBC, cell/ mm^3^	779-7202^*^	<10
Lymphocytes, %	88	
MBP, ng/ml	>139	<6.2

Cerebrospinal fluid analysis

The analysis of cerebrospinal fluid (CSF) cells revealed a low white blood cell count, predominantly comprising lymphocytes. Additionally, CSF protein analysis indicated markedly elevated levels of CSF myelin basic protein, as detailed in Table 1. Moreover, the presence of positive oligoclonal bands was noted in the CSF, which were absent in the corresponding serum sample.

CNS imaging

**Figure 1:**
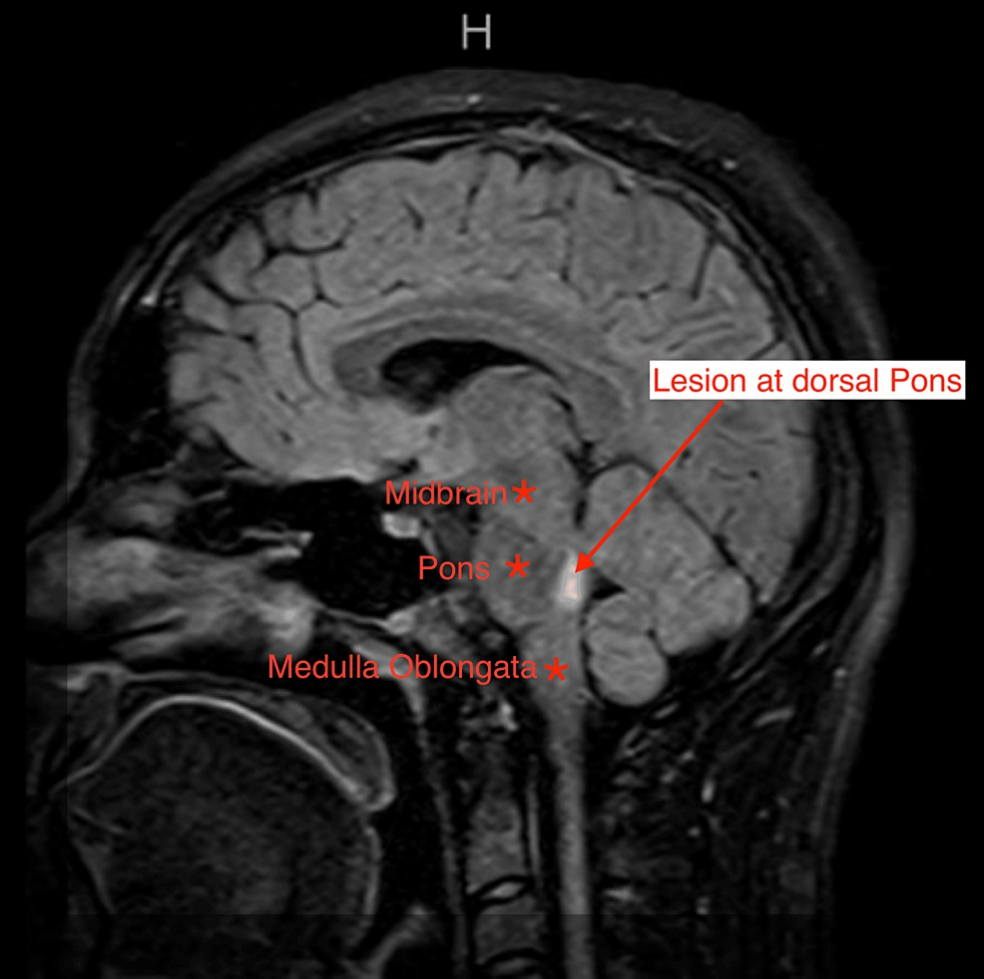
MRI brain FLAIR-weighted image lesion in sagittal view Sagittal view MRI brain FLAIR-weighted image depicting a hyperintense lesion located at the level of the dorsal pons (indicated by the red arrow). The lesion appears as a bright area against the surrounding tissue, suggestive of increased fluid content or inflammation MRI: magnetic resonance imaging; FLAIR: fluid-attenuated inversion recovery

**Figure 2:**
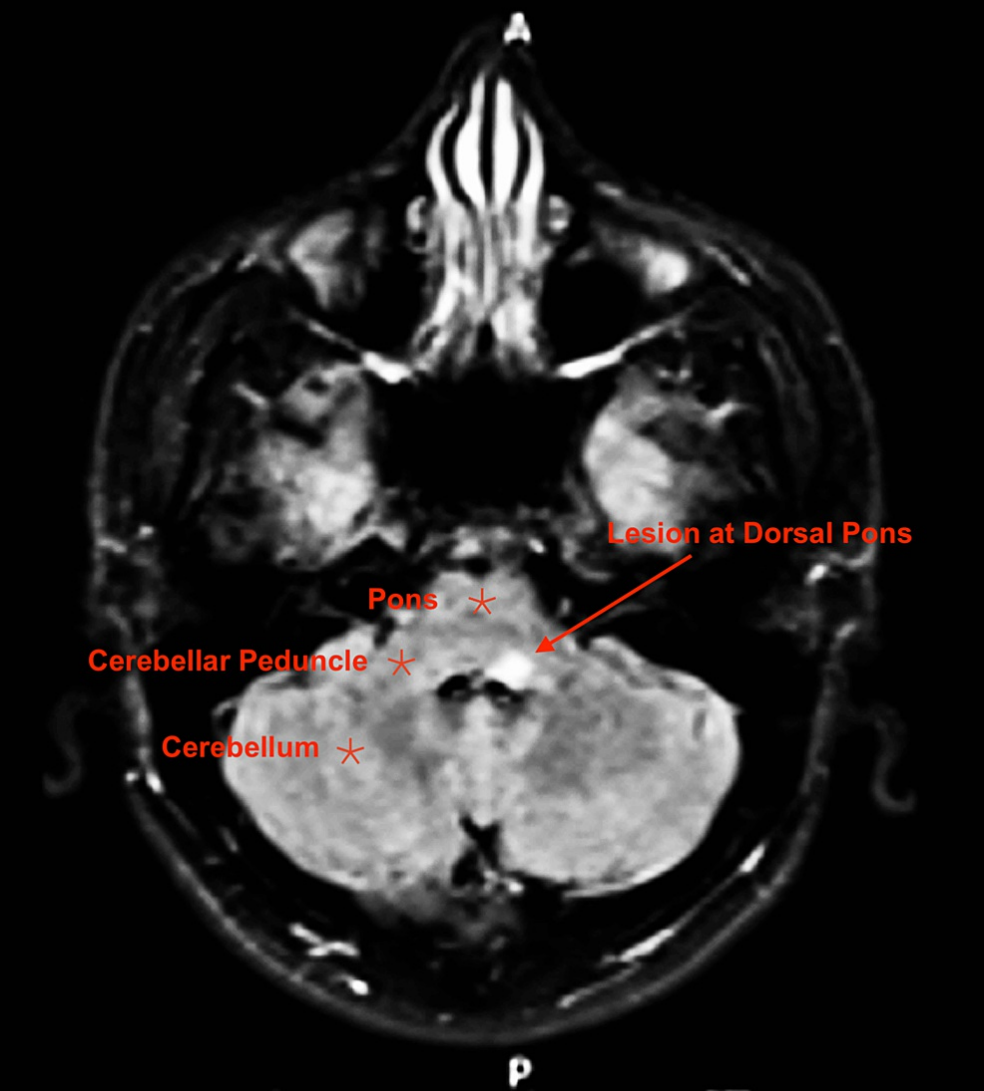
MRI brain FLAIR-weighted image in axial view Axial view MRI brain FLAIR-weighted image displaying a hyperintense lesion situated at the level of the middle cerebellar peduncle. The lesion is depicted as a bright area against the surrounding tissue, suggestive of a potential pathology such as inflammation or demyelination MRI: magnetic resonance imaging; FLAIR: fluid-attenuated inversion recovery

**Figure 3:**
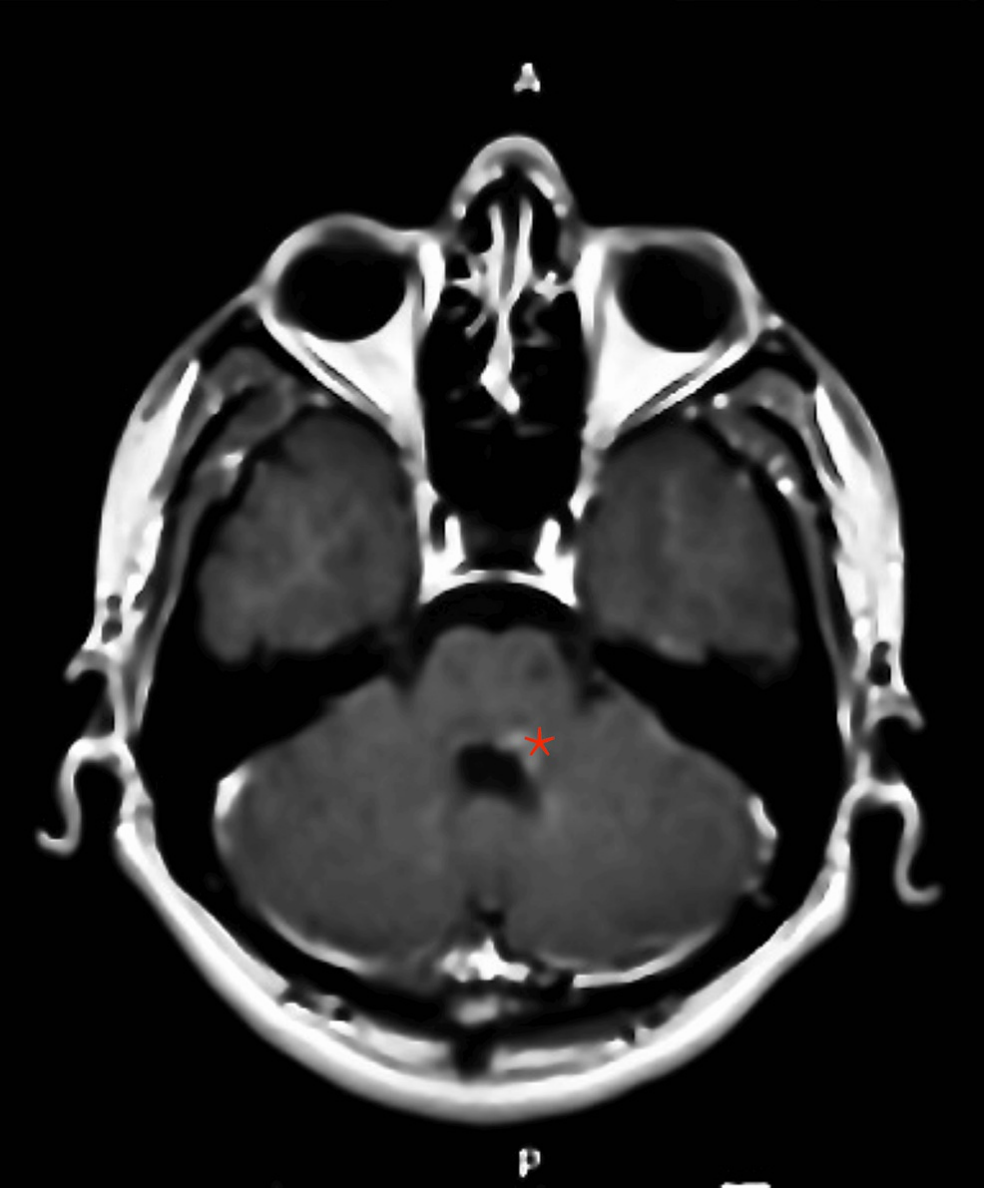
Contrast-enhanced MRI in axial view at the level of middle cerebellar peduncle Contrast-enhanced MRI displaying patchy abnormal enhancement on the left side of the dorsal pons, extending to the left brachium pontis (indicated by a red asterisk) MRI: magnetic resonance imaging

The CT scan of the brain revealed no signs of intracranial hemorrhage or mass effect. However, MRI without contrast showed hyper-intensity on T2/FLAIR sequences in the left pons, potentially indicating a mass effect (Figures 1-2). Subsequent contrast-enhanced MRI revealed patchy abnormal enhancement on the left side of the dorsal pons, extending to the left brachium pontis, suggesting a possible active demyelinating lesion, with acute disseminated encephalomyelitis considered less likely (Figure 3). Additionally, a potential small chronic demyelinating focus was identified in the left temporal white matter. MRI of the spinal cord demonstrated no abnormal signals or enhancements in the spinal canal or neural foramina, effectively ruling out central spinal canal or neural foramina stenosis.

Treatment

Despite the patient's presentation with a solitary clinical episode, a diagnosis of multiple sclerosis was inferred based on the MRI findings and the detection of CSF-specific oligoclonal bands. Accordingly, the patient was started on a regimen of high-dose intravenous steroids: Solu-Medrol 1000 mg over five days.

Follow-up

Following the initial intervention, the patient was started on biologic therapy with beta interferon and a tapering regimen of steroids. He was advised to follow up with a neurologist as an outpatient. Over subsequent visits, the patient demonstrated a gradual improvement in his symptoms, indicating a slow but positive response to the treatment regimen. Close monitoring and continued neurological follow-up were recommended to ensure ongoing management and assessment of the patient's progress.

## Discussion

Distinguishing between Bell’s palsy and MS remains a clinical challenge, particularly due to their overlapping symptoms. Bell’s palsy, typically involving the peripheral nervous system, presents as acute unilateral facial paralysis. On the contrary, MS primarily affects the CNS, often manifesting as visual disturbances, paresthesia, impaired coordination, and potential bladder dysfunction and ataxia [1]. The conventional understanding of Bell's palsy involves lower motor neuron facial palsy impacting both upper and lower facial regions, distinguishing it from central causes primarily affecting the lower facial muscles [2,3]. However, various studies indicate that peripheral facial palsy can paradoxically result from central lesions involving the facial nucleus or nerve at the pons [2,3].

In our case, the diagnostic challenge originated from the reliance on pattern recognition associated with Bell's palsy. This emphasizes the need to be cautious and not let the recognition of typical Bell's palsy symptoms overshadow a thorough evaluation. The central symptoms, which are crucial for distinguishing underlying conditions like MS, might be downplayed by either the patient or the examiner. In this context, the absence of typical MS symptoms such as blurred vision, paresthesia, and clear time/space symptom dissemination added complexity in terms of suspecting MS. This underscores the critical importance of conducting a comprehensive medical history and a thorough neurological examination, ensuring that clinicians do not solely rely on pattern recognition and remain vigilant for subtle signs that may point to alternative diagnoses [4,5].

It is crucial to recognize that both MS and Bell’s palsy can share certain clinical features while often differing in their underlying pathophysiology. The latest guidelines for diagnosing MS underline the importance of considering other neurological signs beyond facial paralysis, such as nystagmus, diplopia, or cerebellar signs, to differentiate between these conditions [6,7]. These additional neurological signs should be thoroughly assessed to establish a comprehensive diagnosis and ensure prompt and appropriate management of these patients [8,9].

Recent advancements in imaging techniques, especially high-resolution MRI, have significantly contributed to differentiating between MS and Bell’s palsy. Imaging plays a pivotal role in revealing distinct lesions and their locations within the CNS [3]. The presence of demyelinating lesions in the brain and spinal cord, as well as specific findings like the "Dawson's fingers" appearance in MRI, can strongly support an MS diagnosis [10].

## Conclusions

This case report highlights the necessity of meticulous clinical assessment in this patient population, by considering a broad spectrum of neurological signs and symptoms beyond facial paralysis. A comprehensive evaluation involving imaging and diagnostic studies remains crucial for accurate diagnosis and management, especially when encountering atypical presentations of CN palsies.
